# Retraction Note: Folic acid and melatonin mitigate diabetic nephropathy in rats via inhibition of oxidative stress

**DOI:** 10.1186/s12986-026-01150-z

**Published:** 2026-06-03

**Authors:** Hossam Ebaid, Samir A. E. Bashandy, Ahmad M. Abdel-Mageed, Jameel Al-Tamimi, Iftekhar Hassan, Ibrahim M. Alhazza

**Affiliations:** 1 Department of Zoology, College of Science, King Saud University, Riyadh, 11451 Saudi Arabia; 2 Department of Pharmacology, Medical Division, National Research Centre, 33 EL Bohouth St., Dokki, , Cairo, 12622 Egypt; 3 Department of Zoology, Faculty of Sciences, Minia University, El-Minia, Egypt

**Retraction Note: Nutrition & Metabolism (2020) 17:6**

10.1186/s12986-019-0419-7

The Editors-in-Chief have retracted this article. After publication, concerns were raised regarding some of the data presented in the figures, specifically:

- Fig. 8b in this Article appears highly similar to Fig. 2b of [1].
- Fig. 8e in this Article appears highly similar to Fig. 2c of [1].

The Editors-in-Chief therefore no longer have confidence in the presented data.

The Authors Hossam Ebaid, Samir A. E. Bashandy, Jameel Al-Tamimi, Iftekhar Hassan and Ibrahim M. Alhazza disagree with this retraction. The Author Ahmad M. Abdel-Mageed has not responded to correspondence regarding this retraction.

## References

[1] Zaki LH, et al. Hypoglycemic and antioxidant effects of *Hibiscus rosa-sinensis* L. leaves extract on liver and kidney damage in streptozotocin induced diabetic rats. Afr J Pharm Pharmacol. 2017;11(13):161–9.

